# Evaluation of Dynamic Spinal Morphology and Core Muscle Activation in Cyclists—A Comparison between Standing Posture and on the Bicycle

**DOI:** 10.3390/s22239346

**Published:** 2022-12-01

**Authors:** José M. Muyor, José A. Antequera-Vique, José M. Oliva-Lozano, Francisco M. Arrabal-Campos

**Affiliations:** 1 Laboratory of Kinesiology, Biomechanics and Ergonomics (KIBIOMER Lab.), Faculty of Education Sciences, University of Almería, 04120 Almería, Spain; 2 Health Research Centre, Faculty of Education Sciences, University of Almería, 04120 Almería, Spain; 3 Engineering Department, University of Almería, 04120 Almería, Spain

**Keywords:** posture, kyphosis, lordosis, sacral tilt, electromyography, cycling

## Abstract

(1) Background: Cycling is characterized by a sustained sitting posture on the bicycle, where physiologic spinal curvatures are modified from standing to cycling. Therefore, the main objective was to evaluate and compare the morphology of the spine and the core muscle activity in standing posture and cycling at low intensity. (2) Methods: Twelve competitive cyclists participated in the study. Spinal morphology was evaluated using an infrared-camera system. Muscle activation was recorded using a surface electromyography device. (3) Conclusions: The lumbar spine changes its morphology from lordosis in standing to kyphosis (lumbar flexion) when pedaling on the bicycle. The sacral tilt significantly increases its anterior tilt when cycling compared to when standing. The spinal morphology and sacral tilt are dynamic depending on the pedal’s position during the pedal stroke quadrants. The infraspinatus, latissimus dorsi, external oblique, and pectoralis major showed significantly higher activation pedaling than when standing, although with very low values.

## 1. Introduction

Cycling is characterized by a sustained sitting posture on the bicycle, in which the cyclist is in contact with three components of the bicycle: seat, handlebar, and pedals [1]. These components, which are adjusted for each individual cyclist, may increase the rider’s peak power output and improve performance [2]. In addition, because aerodynamic drag is a crucial variable that affects cycling performance [3], the design and geometry of bicycles and their components need to be set so that the cyclist is positioned at a greater angle of trunk inclination. These adjustments minimize aerodynamic drag while cycling [4], even if the upper body is in an unnatural position [1].

When the thoracic kyphosis and lumbar lordosis are within a normal range of angular values, there is a lower risk of back pain [5]. In addition, spinal modifications on the sagittal plane’s physiologic curvatures have been associated with spinal disorders [6]. For instance, an increased thoracic kyphosis, lumbar inversion, or a trunk inclination produces greater intradiscal pressure [7], tension in the passive elements of the spine [8], and creep in the lumbar viscoelastic structures [9].

In this regard, several studies have evaluated cyclists’ posture on the bicycle [4,10,11,12,13,14,15,16,17,18,19,20,21,22,23,24,25,26]. Many of these studies have used static evaluation techniques, such as radiographs [13,24] or the Spinal Mouse system [15,16,17,18,19,20,21]. Other studies have evaluated the dynamic morphology of the spine for several seconds while the cyclist pedals [10,11,12,22,23,25,26]. However, most showed mean spinal values in a specific posture or cycling measurements based on static postures without providing information on potentially optimum joint angles based on dynamic assessment [27], and none of the studies evaluated the dynamic spinal morphology in relation to the pedal position.

In addition to the posture adopted by the cyclist on the bicycle, another characteristic of cycling is the need to maintain balance and generate power on the pedals in order to displace the bicycle [28], where the trunk angle influences muscle recruitment and intermuscular dynamics in the lower limbs [29]. In this regard, core stability is crucial in efficiently transferring power from the lower to the upper body and vice versa and maintaining proper body position for more extended periods [30].

Because the majority of studies assessing cyclist posture used static techniques, despite cycling being a dynamic (cyclical) sport, and some of them have evaluated the muscle activity in a cycloergometer and not on road bicycles, the main aims of the current study were: (1) to evaluate and compare the morphology of the thoracic and lumbar spine and sacral tilt in the sagittal plane in standing versus pedaling on a road bicycle (intra-cycle change); (2) to evaluate and compare the muscle activity of the trapezius (upper and middle fibers), infraspinatus, latissimus dorsi, erector spinae, anterior rectus abdominis, external oblique, and pectoralis major under these conditions, in standing versus pedaling on a road bicycle; and (3) to correlate spinal morphology and muscle activity in standing posture and pedaling on a road bicycle.

## 2. Materials and Methods

### 2.1. Participants

A total of twelve competitive and healthy male cyclists (age: 39.91 ± 11.48 years; BMI: 23.85 ± 3.20; training experience: 9.16 ± 8.52 years; and total distance per year: 11,075 ± 4458 km) without current pain whilst cycling, voluntarily participated in the study.

The inclusion criteria for the participants were: (1) daily cycling training of between two and four hours, (2) three to five training days per week, and (3) at least three years of training experience. The exclusion criteria were (1) a history of spinal pain in the six months before the study, (2) a history of spinal surgery, (3) a medically diagnosed spinal disorder or evidence of any kind of limitation during the current study. All participants were instructed to avoid physical activity 24 h before the study. The Bioethical Committee of the University of Almería authorized this study (Ref: UALBIO2022/025) under the ethical principles of the Declaration of Helsinki. Written signed consent was obtained from each participant before taking part in the study.

### 2.2. Procedure

#### 2.2.1. Electromyography (EMG) Setup and Data Collection

Prior to undertaking the tests, the participants’ skin was prepared for the placement of electrodes for the recording of electromyographic signals to exclude any influence of electrical noise. In order to do this, hair was removed (shaved) from the parts of the body where the electrodes would be placed. These areas were then cleansed with cotton and 96 percent alcohol, then softly abraded with fine sandpaper. Bipolar adhesive Ag/AgCl electrodes (Medico Lead-Lok, Noida, India) were then positioned under the manufacturer’s instructions, spacing the electrode pairs 2 cm apart and placing the reference electrode distant from the electrode pair.

On the right side of the trunk, the electrodes were positioned (participant’s dominant side of the upper and lower limbs), using the spinal column as the middle part and under the recommendations for the Surface Electromyography for the Non-invasive Assessment of Muscles (SENIAM) [31]. A detailed description of electrode placement is shown in Table 1.

After placing the electrodes, the maximal voluntary isometric contraction (MVIC) of each muscle was recorded to normalize the electromyography (hereafter EMG) values registered and to compare the % MVIC while standing posture versus cycling at 90 watts. Two sets of 3-s MVIC randomized trials were recorded for each muscle, with a 2-min gap between each MVIC assessment and a rest period of around 10 s in between each contraction [39]. The peak EMG value (50 ms window) recorded during the MVIC was used to determine the MVIC. Table 1 displays the MVIC maneuver for each muscle.

Using a WBA Mega device (Bittium Biosignals, Kuopio, Finland), EMG signals for each muscle were captured and sampled at 1000 Hz. An A/D converter (National Instruments, New South Wales, Australia) was used to convert the analog signal to a digital one. LabView software (National Instruments, Austin, TX, USA) was then used to filter the digital signal by bandwidth (12–450 Hz) using a fourth-order Butterworth filter. From the raw EMG signals, RMS signals were computed by applying 50 ms sliding windows with the MEGAWIN software program (Bittium Biosignals, Kuopio, Finland) for further analysis.

#### 2.2.2. Motion Capture

**Figure 1:**
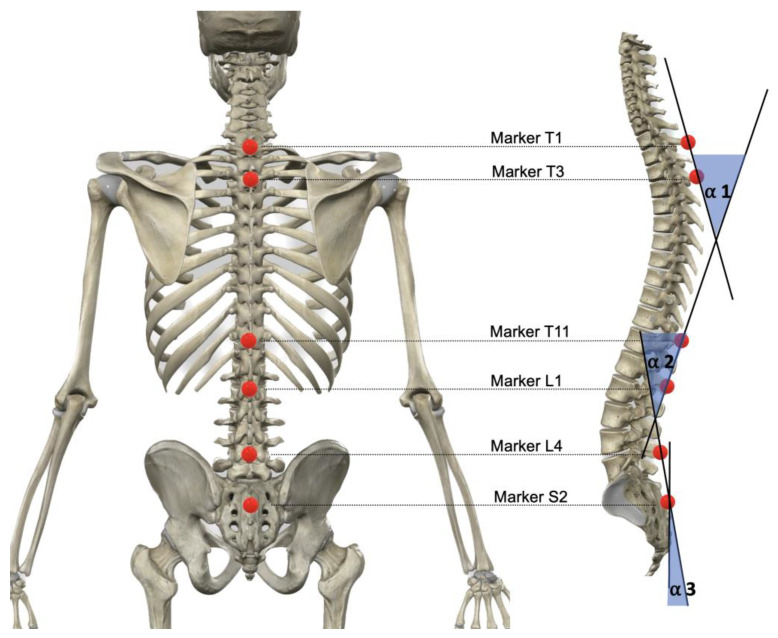
Placement of markers and definition of calculated angles.

In the sagittal plane, spinal morphology (thoracic and lumbar curvatures) and sacral tilt were evaluated using a sixteen-infrared-camera system (Flex 3, Optritrack, Natural Point, OR, USA), calibrated according to the manufacturer’s specification, with a sampling frequency of 100 Hz. Six spherical reflective markers (B&L Engineering, Tustin, CA, USA) were attached to specific anatomic landmarks of the participants in a standing posture. One marker was positioned at each spinous process as follows: on the first and third thoracic vertebrae (T1 and T3), on the eleventh thoracic vertebrae and the first lumbar vertebrae (T11 and L1), and on the fourth lumbar vertebrae and the second sacral vertebrae (L4 and S2) (Figure 1). The thoracic curvature angle was calculated in the sagittal plane using the angle between the segments of the spine defined by the markers T1-T3 and T11-L1 (α 1), the lumbar curvature angle was calculated using the angle between the segments defined by the markers T7-L1 and L4-S2 (α 2), and the sacral tilt was calculated using the angle between the segments defined by the markers L4-S2 and the vertical plane (α 3) (Figure 1). This methodology and technology of motion capture have been reported as a valid and reliable system for measuring thoracic and lumbar spinal curvatures and sacral inclination in research and clinical environments [40]. Two additional markers were placed on each pedal of the bicycle to identify each pedaling cycle [26], which would be necessary for future analysis of spine morphology and sacral tilt on the bicycle. All datasets are based on the averages of 30-s intervals of standing on the floor (for assessing the standing posture) and 30 s of pedal strokes of the dominant leg at 90 watts (to assess cycling on a road bicycle).

No gap filling or filtering routines were performed on the data before they were outputted to an ASCII file for analysis. Following the procedure established by Muyor et al. [40], this ASCII file was loaded into Matlab^®^ software (The MathWorks, Natick, MA, USA) to calculate the thoracic and lumbar angles and sacral tilt, following the equations [40].

##### Standing

The measurements of standing posture were performed on cyclists who were wearing a culotte and standing barefoot on the floor in a natural (relaxed) position with their eyes and ears in line with the horizontal, their arms at their sides, their knees almost fully extended, and their feet shoulder-width apart. Cyclists were required to remain in this natural standing position for 30 s to capture spinal morphology.

##### On the Bicycle

Cycling shoes and culottes were worn by the cyclists. They rode on their personal bike. The participants used a cycling trainer (PowerBeam ProTrainer ANT+, CycleOps, Madison, WI, USA) while sitting and pedaling for five minutes at a cadence of 90 revolutions per minute. Most laboratory investigations have already stated that this tempo is the most cost-effective [15,41]. The cycle resistance was programmed to 90 watts; this resistance was deemed very light to avoid the possible influence of more intense resistances on the spinal morphology. Cyclists were required to pedal for five minutes, of which the first 30 s of minute four (from 00:04:00 to 00:04:30) were captured to evaluate the spinal morphology. Specifically, 45 pedal strokes (i.e., 45 pedaling cycles) were recorded for each cyclist. For the calculation of spinal curvatures and sacral tilt, to each cyclist was individually identified each one of their pedaling cycles. Subsequently, the mean values of the 45 pedal strokes were calculated for each one of the spinal curvatures and sacral tilt in each one of the quadrants of the pedal stroke (0°, 90°, 180°, and 270°).

### 2.3. Statistical Analysis

Firstly, the hypotheses of normality and homogeneity of variances were analyzed using the Shapiro–Wilk test and demonstrated that all data had a normal distribution (*p* > 0.05). For the statistical analyses, means and standard deviations were calculated for all variables. Each spinal morphology of the curvatures (thoracic and lumbar) and sacral tilt and each muscle (upper trapezius fibers, middle trapezius fibers, infraspinatus, latissimus dorsi, erector spinae, anterior rectus abdominis, external oblique, and pectoralis major) were compared separately in both the standing position and pedaling on the road bicycle. A dependent t-test for both postures (standing and pedaling on the bicycle) was used for each spinal curvature and muscle as appropriate for the repeated measure. Correlations between two parameters (spinal morphology and muscle activity) were determined using the Pearson correlation coefficient.

The data of the thoracic spine, lumbar spine, and sacral tilt in each pedal stroke quadrants were statistically tested using a one-way ANOVA with repeated measures. Wilk’s lambda, Pillai’s trace, Hotelling’s trace, and Roy’s tests, which all produced comparable findings, all confirmed the significance of the repeated multivariate measurements. Additionally, Mauchly’s test of sphericity was run to evaluate the variance hypotheses. If an assumption was broken, a Huynh–Feldt adjustment was performed to change the degrees of freedom. A post-hoc Bonferroni test for multiple comparisons was performed if a significant *p*-value was found for the main effect of the ANOVA. Partial eta-squared (η^2^_p_) was used to estimate the explained variance and effect size.

The effect size was calculated through Cohen’s d using the combined standard deviation formula [42]. An effect size of d > 0.8 was considered large, while d at approximately 0.5 was considered moderate, and d < 0.2 was considered small [42]. The statistical power and effect sizes were calculated using G*power 3.1 for Mac OS X [43]. Statistical analyses were carried out using the IBM SPSS software (v.27), and an alpha level of 0.05 was used for all statistical tests.

## 3. Results

**Figure 2:**
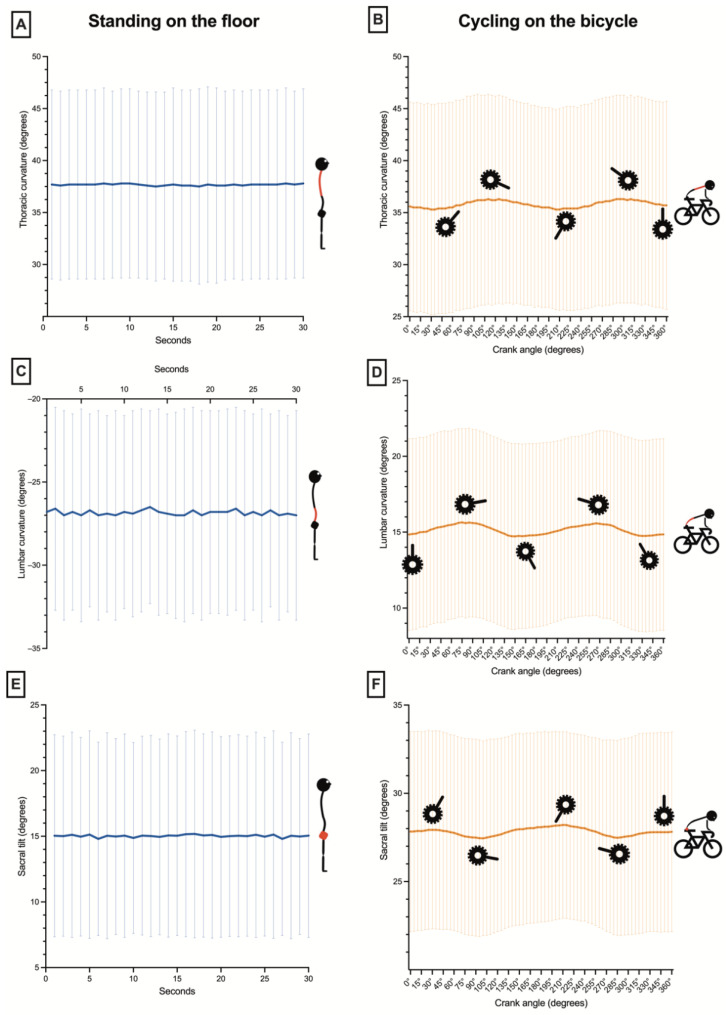
Average angles and standard deviation of the spinal curvatures and the sacral tilt, for the 12 cyclists, in standing on the floor and pedaling on the road bicycle.

The average angles of the thoracic curvature in standing and pedaling on the road bicycle are shown in Figure 2A,B. The thoracic spine in standing posture (Figure 2A) presented higher values (mean = 37.71° ± 9.43°) than on the bicycle (35.82° ± 9.91°) (Figure 2B), although without statistically significant differences and with a small effect size (*p* = 0.51; *d* = 0.20). A thoracic flexion–extension was observed during the pedaling cycle. It was greater at 110° and 310°and lower at 45° and 210° crank of the pedaling cycle (Figure 2B).

**Figure 3:**
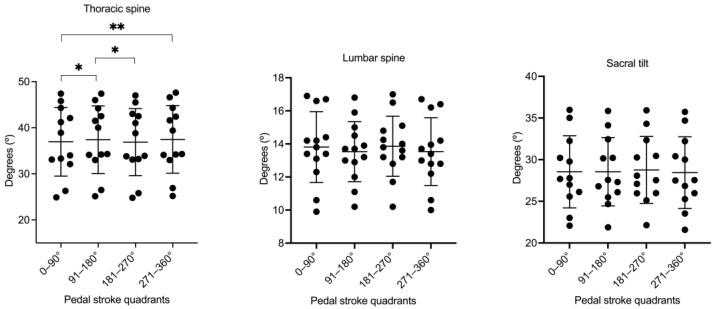
Comparisons of the spinal morphology in each pedal stroke quadrant. The dots represent the mean value at a given quadrant. * *p* < 0.05; ** *p* < 0.01.

ANOVA showed significant changes in the thoracic flexion according to the crank angle (F_(3,30)_ = 8.64, *p* < 0.001, η^2^_p_ = 0.46). Post-hoc comparisons revealed significant greater thoracic flexion in the second and fourth pedal stroke quadrants with respect to the first and third quadrants (Figure 3).

The lumbar curvature in standing posture was in lordosis (negative values; mean = −26.82° ± 6.36°) (Figure 2C). However, when pedaling on the bicycle, the lumbar spine was flexed (positive values; mean = 15.14° ± 6.14°) (Figure 2D). The differences of these lumbar values were statistically significant between both postures (standings vs. pedaling) with a large effect size (*p* < 0.001; *d* = 1.92). A lumbar flexion–extension was observed during the pedaling cycle. It was greater at 75° and 270° and lower at 0° and 160° of the pedaling cycle (Figure 2D). However, ANOVA did not show significant changes in the lumbar flexion according to the crank angle (F_(1_._30,13_._01)_ = 2.04, *p* = 0.16, η^2^_p_ = 0.17) (Figure 3).

The sacral tilt in standing posture (Figure 2E) showed significantly lower values than pedaling on the bicycle (15.13° ± 7.88° vs. 27.81° ± 5.47, respectively; *p* < 0.001) with a large effect size (*d* = 2.43). The greater anterior sacral tilt was at 30° and 215°, and the lower sacral tilt was at 100° and 290° (Figure 2F). However, ANOVA did not show significant changes in the sacral tilt according to the crank angle (F_(1_._25,12_._55)_ = 1.70, *p* = 0.18, η^2^_p_ = 0.14) (Figure 3).

**Figure 4:**
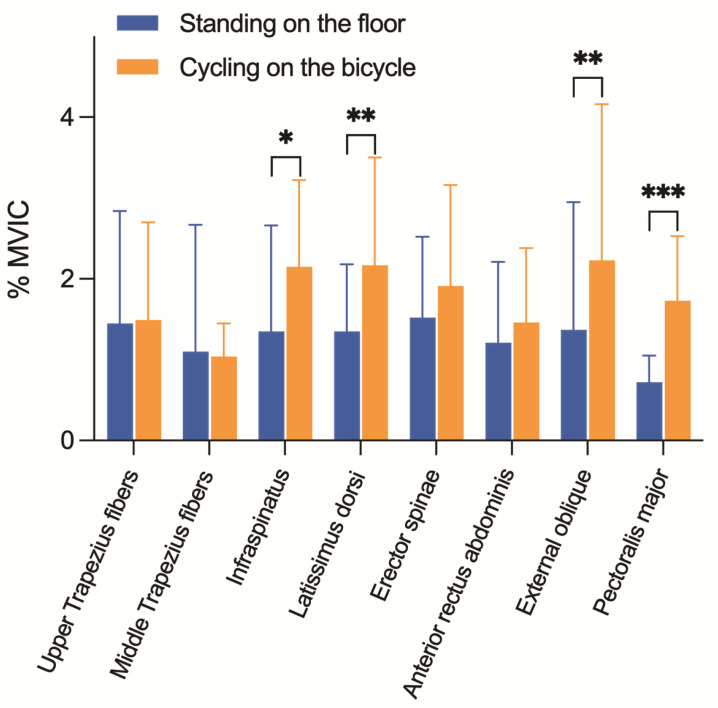
Comparison of the muscle activity, expressed as a percentage of the maximal voluntary isometric contraction (% MVIC), in a standing position versus on the bicycle. * *p* < 0.05; ** *p* < 0.01; *** *p* < 0.001.

Figure 4 shows a comparison of the muscle activity, expressed as a percentage of the maximal voluntary isometric contraction (% MVIC) in both postures analyzed (standing versus pedaling on the bicycle at 90 watts). There was significantly greater activation for the infraspinatus (*p* = 0.05, *d* = 0.6), latissimus dorsi (*p* = 0.01, *d* = 0.8), external oblique (*p* = 0.03, *d* = 0.7), and pectoralis major (*p* ≤ 0.001, *d* = 1.7) when pedaling on the bicycle than when standing.

In the standing posture, mean angular values of the thoracic curvature showed a positive and statistically significant correlation with the mean angular values of the thoracic curvature pedaling on the road bicycle (*r* = 0.746; *p* = 0.008) and a negative and statistically significant correlation with the mean angular values of the sacral inclination pedaling on the road bicycle (*r* = −0.680; *p* = 0.021). In addition, in standing posture, mean angular values of the sacral inclination showed a positive and statistically significant correlation with the mean angular values of the lumbar curvature (*r* = 0.847; *p* < 0.001) and a positive and statistically significant correlation with the mean angular values of the sacral inclination pedaling on the road bicycle (*r* = 0.725; *p* = 0.012).

In the pedaling position on the bicycle, mean angular values of the thoracic curvature showed a negative and statistically significant correlation with the mean angular values of the sacral inclination (*r* = −0.618; *p* = 0.043) and a negative and statistically significant correlation with the muscle activity of the pectoralis major (*r* = −0.832; *p* = 0.005). In addition, the muscle activity for the upper trapezius fibers showed a positive and statistically significant correlation with the muscle activity for the erector spinae (*r* = 0.933; *p* < 0.001).

No other statistically significant correlation was found among all the other variables analyzed in the current study.

## 4. Discussion

One of the primary purposes of the current study was to evaluate and compare the morphology of the thoracic and lumbar spine and sacral tilt in the sagittal plane when in a standing posture versus when cycling on a bicycle. To the best of our knowledge, this is one of the first studies to analyze the dynamics of spinal morphology. There is a widespread belief that, in cycling, the spine maintains a static position, possibly because, to the naked eye, there are no perceived changes in its dynamics, depending on the pedal’s position, in the pedaling cycle. In this regard, it was observed that both when standing and pedaling on the bicycle, the morphology of the spine is not static. We found slight movements in thoracic kyphosis, lumbar lordosis, and sacral inclination in the standing posture. These movements are possibly due to the breathing of the participants and their motor control when maintaining a stable posture. The observation of these values reflects the precision of the evaluation system used in this study. Cyclic spinal movements (thoracic, lumbar, and sacral tilt) were observed on the bicycle, clearly related to the pedaling cycle.

Another widespread belief is that cycling generates a significant increase in thoracic kyphosis. In part, this could be due to the perception that the predominant posture in cycling involves the flexing of the trunk to reach the handlebars [1]. Previous studies have reported that in those sports where the dominant posture involves trunk flexion, the athletes show thoracic hyperkyphosis [44,45]. In the current study, when comparing both postures (standing vs. cycling on the bicycle), the results showed that although the thoracic curvature’s angular values were higher than on the bicycle, these differences were not statistically significant. However, the lumbar spine changed from lordosis (anterior convexity) in the standing posture to kyphosis (posterior convexity) when cycling on the bicycle. In addition, it showed a significantly greater inclination of the sacrum when on the bicycle than when standing.

Muyor et al. [17] found a significantly greater thoracic kyphosis in elite cyclists than in non-athlete participants in a standing posture. However, they did not find any statistical differences in the thoracic kyphosis as the grip on the handlebars was more distal and lower in relation to the saddle height. The present study adds to the existing literature on how the thoracic spine increases and decreases its angular values depending on the pedal’s position. Moreover, there seem to be three cyclist clusters in the spinal curvatures and the sacral tilt. Possibly, this differentiation between the postures adopted by the cyclists is due to the adjustment of the bicycle itself in search of greater comfort or aerodynamics. Thus, each cyclist would have configured their bicycle according to their trunk flexion capacity (thoracic and lumbar) and sacral inclination. In this sense, it should be remembered that each cyclist was evaluated on the posture adopted on their own bicycle.

Regarding the lumbar spine in cyclists, Usabiaga et al. [24] were among the first researchers to report the modification from lumbar lordosis in the standing posture to lumbar kyphosis on the bicycle. Subsequently, other studies have drawn similar conclusions, finding greater vertebral flexion as the grip on the handlebars is more distal and lower in relation to saddle height. In contrast, there were only statistically significant differences in greater lumbar flexion when the handlebar grip was more aerodynamic (time trial) [15,21]. However, previous studies seem to agree that sacral tilt is significantly greater the lower the handlebar’s height is relative to the saddle [15,19,21].

As in the thoracic curvature, the present study reported cyclical flexion–extension movements of the lumbar spine (although inverted). There were also fluctuating values of the inclination of the sacrum (approximately ~1°), depending on the pedal’s position during the pedaling cycle. Therefore, it could be assumed that these spinal movements and the tilt of the sacrum are inherent to the pedaling action itself. This spinal motion would contribute to a transfer of power from the upper to the lower body through the reaction forces of the hip joint [46].

Another of the main objectives of the present study was to evaluate and compare the electromyographic activity of the trunk musculature in the standing posture versus cycling on the bicycle. The results showed significantly greater activation for the infraspinatus, latissimus dorsi, external oblique, and pectoralis major, pedaling on the bicycle at 90 watts than when in the standing posture. However, there were no statistical differences for upper and middle trapezius fibers, erector spinae, and anterior rectus abdominis. In this regard, Abt et al. [28] reported that core stability contributes to lower extremity cycling mechanics. These improvements in core strength could promote greater torso stability within the saddle and maintain lower extremity alignment to apply greater force transmission to the pedals.

In developing this study, we selected the trunk musculature that was considered most important for maintaining upper body posture on the bicycle. Moreover, to avoid the inclusion of any pain variable that could affect the activation of muscles or posture adopted by the cyclists, it was decided only to evaluate healthy cyclists in the study. In this regard, Burnett et al. [11] reported that, compared to healthy control samples, cyclists who reported low back pain showed a trend towards increased lower lumbar flexion and rotation with an associated loss of co-contraction of the lower lumbar multifidus. In this regard, it is worth noting the low muscle activation found when pedaling at 90 watts (<2.5% MVIC). Moreover, when pedaling, half of the analyzed muscles showed a muscle activation similar to that found when standing upright (~1.5% MVIC). Kuo and Zajac [47] reported that in the standing posture, the body has several multi-joint movement strategies for controlling the center of mass with minimal muscle activation (called “neural effort”). This strategy is possibly due to the body trying to be as efficient as possible, activating the musculature as little as possible to maintain the standing posture. Given our results and the low muscle activation found while pedaling on the bicycle, we could consider that this theory of minimum activation efficiency could also be applied to cyclists. The significantly greater level of muscle activation found on the bicycle (approximately 1% MVIC) than when standing could be justified by these four muscles’ postural/stabilizing function. In addition, because there was such a low muscle activation, no different patterns were observed in the activation of the muscles evaluated in each quadrant of the pedaling cycle. 

The current study has several limitations that should be considered in future research. The first is the evaluation of spinal morphology at low pedaling intensity (90 watts). This intensity was selected to compare the cyclist’s spinal posture simply by pedaling on the bicycle, vis-à-vis the posture adopted in the standing position, without being influenced by the resistance to be overcome during pedaling. The current study can serve as a baseline for understanding how spinal morphology and core muscle activation adapt from standing to cycling posture. However, in competitive cycling, much higher pedaling intensities are produced. Future studies should analyze the spinal morphology adopted according to different intensity zones (cardiovascular) or pedaling watts. In addition, another limitation was evaluating the dynamic morphology of the spine without considering the effect of fatigue. Cycling is a long-duration sport, so it would be interesting for future studies to analyze the impact of fatigue on the behavior of spinal morphology. In addition, another limitation was the evaluation of the percentage of muscle activation based on the maximum voluntary isometric contraction (MVIC). This choice was selected by taking into consideration the methodology developed by previous studies. However, cycling is a sport characterized by dynamic and cyclical pedaling, not isometric contraction. Thus, future studies could compare the degree of activation of the trunk musculature, considering the muscle contraction achieved during a maximal effort in cycling as opposed to using a maximal isometric contraction.

## 5. Conclusions

Although the mean values of the thoracic flexion were greater in standing than on the bicycle, there were no significant differences between both postures. The lumbar spine changes its morphology from lordosis in standing to kyphosis (lumbar flexion) when pedaling on a bicycle. The sacral tilt significantly increases its anterior inclination when cycling than when standing. Moreover, the spinal morphology and sacral tilt are dynamic (changing their angular values cyclically) depending on the pedal’s position during the pedal stroke quadrants. Nevertheless, although there was a sinusoidal behavior of the spinal curvatures and sacral angles, the range was very low, around 1°. On the other hand, when pedaling on a bicycle, the infraspinatus, latissimus dorsi, external oblique, and pectoralis major showed significantly higher activation than when standing, although with very low values.

## Figures and Tables

**Table 1:** Surface electrode placement and maximal isometric voluntary contraction (MVIC) maneuver description.

Muscle	Electrode Placement	MVIC Maneuver
Upper Trapezius fibers	At 50% of the line from the acromion to the spine on vertebra C7 [32].	In a standing position, the cyclists engaged in a scapular elevation and abduction while facing manual resistance in the contrary direction.
Middle Trapezius fibers	Approximately halfway between the medial border of the scapula and the spine, at the level of the vertebra T3 [32].	In a standing position, the cyclists performed a scapular elevation and abduction while facing manual resistance in the contrary direction.
Infraspinatus	At 50% of the scapula’s spine, over the infrascapular fossa of the scapula, laterally, at 50% of the line from vertebra T6 to the greater tubercle of the head of the humerus [32].	Shoulder externally rotated and abducted at 90°, and elbow flexed at 90°. The cyclists performed an isometric contraction of the shoulder external rotators.
Latissimus dorsi	At 4 cm below the inferior tip of the scapula, half the distance between the spine and lateral edge of the torso, with an oblique angle of ~25° [33].	In a standing position, with shoulders and elbows flexed to 90° (in the horizontal plane), the cyclists performed scapular-humeral adduction, which involves pushing against manual resistance to move the humerus closer to the trunk.
Erector spinae	Laterally, about 2 cm from the vertebra L3 [34].	Lying face down on a stretcher, cyclists were strapped in their lower limbs with their trunks unsupported. Cyclists maintained a steady posture with their trunks parallel to the ground as manual resistance was supplied via downward pressure at the mid-thoracic vertebrae region [35].
Anterior rectus abdominis	At 3 cm laterally to the midline and midway between the xiphoid process and the umbilicus [36].	Cyclists were required to perform a resisted curl-up exercise [37].
External oblique	Above the anterior superior iliac spine at an oblique angle, at the level of the umbilicus [36].	Cyclists were supine in a hook-lying position with their feet flat on the floor. With manual resistance given at the shoulders in the direction of trunk extension and correct rotation, the trunk was fully flexed and rotated to the opposite side of the manual resistance [37].
Pectoralis major	Over the fifth intercostal gap on the midclavicular line [38].	The cyclists had to push opposing manual resistance moving the other way while standing with their shoulders and elbows extended at 90 degrees (in the horizontal plane) to mimic the pec-deck workout.

## Data Availability

Not applicable.
